# Supplementary material for: Metabolomic analysis of the occurrence of bitter fruits on grafted oriental melon plants
Source: PLoS One. 2019 Oct 10;14(10):e0223707. doi: 10.1371/journal.pone.0223707 (PMC6786619; doi:10.1371/journal.pone.0223707)
Supplement: S1 Table — (DOC) [file pone.0223707.s001.doc]

**Table S1 Information of different rootstocks**

| **Rootstocks** | **Source of different rootstocks** |
| --- | --- |
| *Cucurbita maxima* Duch.var.Yemuyixiong | Delifengtian agricultural technology company of Beijing |
| *Cucurbita maxima* Duch.var.Ribenxuesong | Hongliang seedlings Ltd. of shandong |
| *Cucurbita maxima* Duch.var.Qingshengzhenjia | Pingjunhongdu rootstock research institute of shandong |
| *Cucurbita maxima* Duch.var.Jingxinzhen3 | Jingyanyinong technology development center of Beijing |
| *Cucurbita maxima* Duch.var.Feichangfuzuo | Yijiuxiang seedlings Ltd. of Shenyang |
| *Cucurbita maxima* Duch.var.Jingyutianzhen1 | Jingyuweier agricultural technology company of Beijing |
| *Cucumis melo* L. (Muskmelon Inbred line1) | Hebei Key Laboratory of Vegetable Germplasm Innovation and Utilization |
| *Cucumis melo* L. (Muskmelon Inbred line2) | Hebei Key Laboratory of Vegetable Germplasm Innovation and Utilization |
