# Supplementary material for: Metabolomic analysis of the occurrence of bitter fruits on grafted oriental melon plants
Source: PLoS One. 2019 Oct 10;14(10):e0223707. doi: 10.1371/journal.pone.0223707 (PMC6786619; doi:10.1371/journal.pone.0223707)
Supplement: S2 Table — (DOC) [file pone.0223707.s002.doc]

**Table S2 Comparison of metabolites in fruits of plants grafted onto Ribenxuesong rootstocks and non-grafted plants**

| **Classification** | **Name** | **Non-grafted** | **Grafted with pumpkin rootstock** | **Content changes** |
| --- | --- | --- | --- | --- |
| Phosphate ester | PS(18:1(9Z)/18:2(9Z,12Z)) | 4617.41±1064.4 | 63.04±13.35** | ↓ |
|  | PS(P-18:0/15:0) | 816.63±197.06 | 47.56±11.28** | ↓ |
|  | PA(19:3(10Z,13Z,16Z)/0:0) | 5.33±1.04 | 0.97±0.17** | ↓ |
|  | PS(22:0/22:1(11Z)) | 14.77±5.01 | 0.14±0.05** | ↓ |
|  | PE(16:1(9Z)/18:2(9Z,12Z)) | 286.61±55.7 | 112.51±55.92** | ↓ |
|  | PS(P-16:0/17:2(9Z,12Z)) | 45.05±10.32 | 17.43±3.91** | ↓ |
|  | PA(15:0/22:6(4Z,7Z,10Z,13Z,16Z,19Z)) | 75.43±11.12 | 30.9±6.16** | ↓ |
|  | PE(18:4(6Z,9Z,12Z,15Z)/18:2(9Z,12Z)) | 31.78±8.95 | 10.16±2.05** | ↓ |
|  | PS(O-16:0/17:2(9Z,12Z)) | 286.61±55.7 | 112.51±55.92** | ↓ |
|  | PE(16:0/18:3(6Z,9Z,12Z)) | 5543.23±1592.94 | 2066.17±321.79** | ↓ |
|  | PE(16:0/18:0) | 10896.54±4110.5 | 1911.73±472.68** | ↓ |
|  | PA(17:1(9Z)/0:0) | 2.98±0.78 | 1.24±0.48** | ↓ |
|  | PG(18:2(9Z,12Z)/16:0) | 7778.91±3789.59 | 1377.46±381.63** | ↓ |
|  | PI(16:0/18:2(9Z,12Z)) | 519.81±179.38 | 187.49±45.43** | ↓ |
|  | PA(18:1(11Z)/18:1(11Z)) | 6832.64±813.07 | 1011.26±267.54** | ↓ |
|  | PI(16:1(9Z)/18:1(11Z)) | 2234.44±859.76 | 712.49±180.19** | ↓ |
|  | PS(18:3(6Z,9Z,12Z)/22:6(4Z,7Z,10Z,13Z,16Z,19Z)) | 604.58±91.62 | 136.89±42.84** | ↑ |
|  | PC(18:2(9Z,12Z)/20:0) | 79.45±8.16 | 167.7±16.31** | ↑ |
|  | PA(16:0/18:1(11Z)) | 1871.9±195.26 | 4189.52±1406.92** | ↑ |
|  | PE(18:3(9Z,12Z,15Z)/22:0) | 289.86±215.21 | 629.65±68.42** | ↑ |
|  | PE(18:0/22:2(13Z,16Z)) | 648.53±120.87 | 1737.73±403.99** | ↑ |
|  | PA(16:0/18:2(9Z,12Z)) | 1818.13±206.4 | 6734.33±1058.84** | ↑ |
| Sterols | 3alpha,7beta,12alpha-Trihydroxy-6-oxo-5alpha-cholan-24-oic Acid | 12.38±2.37 | 8.70±1.76* | ↓ |
|  | 7alpha-Hydroxy-3,12-dioxochola-1,4-dien-24-oic Acid | 1.76±0.47 | 1.14±0.29* | ↓ |
|  | 6alpha,7alpha-Dihydroxy-3-oxo-5beta-cholan-24-oic Acid | 13.36±3.13 | 20.54±4.9* | ↑ |
|  | Hippuristanolide | 0.45±0.06 | 13.97±6.84** | ↑ |
|  | 3-O-(Glcb)-6-O-(Glcb)-(25R)-5alpha-spirostan-3beta,6alpha,23S-triol | 130.68±9.56 | 167.17±10.51* | ↑ |
|  | 1beta,3beta,5alpha,6beta-tetrahydroxyandrostan-17-one | 0.09±0.02 | 0.52±0.16** | ↑ |
| Flavonoids | Kaempferol 3-glucosyl-(1->3)- rhamnosyl-(1->2)-[rhamnosyl-(1->6)-galactoside] | 6.94±2.45 | 2.23±0.44** | ↓ |
|  | Pelargonidin 3-(6''-malonylglucoside) -5-glucoside | 28.62±6.38 | 46.16±8.99** | ↑ |
|  | 5,7,3',4'-Tetrahydroxy-3,6,5'-trimethoxyflavone | 6.53±2.00 | 9.53±1.99* | ↑ |
|  | Malvidin 3-rutinoside | 0.00±0.00 | 40.35±6.96** | ↑ |
| Cucurbitacin | Cucurbitacin O | 0.78±0.15 | 312.16±54.46** | ↑ |
|  | Cucurbitacin S | 19.95±3.90 | 2018.90±211.83** | ↑ |
|  | Cucurbitacin C | 9.32±0.98 | 4568.85±1192.68** | ↑ |
| Other compounds | Spiramycin | 34.87±12.88 | 59.34±6.45** | ↑ |
|  | Monoisobutyl phthalic acid | 23.09±3.11 | 27.82±1.45** | ↑ |

Asterisks indicated significant differences (Student t-test: *, P < 0.05; **, P < 0.01) relative to the non-grafted plants. “↑” and “↓” indicated that the fruit metabolite was up- and down-regulated in grafted plants with pumpkin rootstock compared to non-grafted plants. Numbers in third and fourth column represent average ± SD.
