# Supplementary material for: Metabolomic analysis of the occurrence of bitter fruits on grafted oriental melon plants
Source: PLoS One. 2019 Oct 10;14(10):e0223707. doi: 10.1371/journal.pone.0223707 (PMC6786619; doi:10.1371/journal.pone.0223707)
Supplement: S3 Table — (DOC) [file pone.0223707.s003.doc]

**Table S3 Comparison of metabolites in fruits of plants grafted onto Ribenxuesong and muskmelon rootstocks**

| **Classification** | **Name** | **Grafted with muskmelon rootstock** | **Grafted with pumpkin rootstock** | **Content changes** |
| --- | --- | --- | --- | --- |
| Phosphate ester | PE(16:0/18:0) | 12768.08±2164.39 | 1911.73±472.68** | ↓ |
|  | PE(14:0/20:2(11Z,14Z)) | 61105.99±8377.61 | 18628.19±7863.72** | ↓ |
|  | PS(O-16:0/17:2(9Z,12Z)) | 362.04±53.88 | 112.51±55.92** | ↓ |
|  | PS(P-16:0/17:2(9Z,12Z)) | 41.11±7.3 | 17.43±3.91** | ↓ |
|  | PS(22:0/22:1(11Z)) | 15.39±5.59 | 0.14±0.05** | ↓ |
|  | PS(18:1(9Z)/18:3(9Z,12Z,15Z)) | 1667.4±291.15 | 736.99±212.39** | ↓ |
|  | PS(P-18:0/15:0) | 151.55±23.68 | 47.56±11.28* | ↓ |
|  | PS(18:1(9Z)/18:2(9Z,12Z)) | 478.23±196.29 | 63.04±13.35** | ↓ |
|  | PI(P-16:0/17:2(9Z,12Z)) | 204.49±39.78 | 125.16±24.67* | ↓ |
|  | PI(16:0/18:2(9Z,12Z)) | 366.7±50.51 | 187.49±45.43* | ↓ |
|  | PA(15:0/22:6(4Z,7Z,10Z,13Z,16Z,19Z)) | 113.27±18.47 | 30.9±6.16** | ↓ |
|  | PA(17:1(9Z)/0:0) | 3.76±1.14 | 1.24±0.48** | ↓ |
|  | PA(18:1(11Z)/18:1(11Z)) | 5934.63±768.35 | 1011.26±267.54** | ↓ |
|  | PA(16:0/18:2(9Z,12Z)) | 2275.6±535.75 | 6734.33±1058.84** | ↑ |
|  | PA(16:0/18:1(11Z)) | 1364.78±206.52 | 4189.52±1406.92** | ↑ |
|  | PA(18:1(9Z)/18:4(6Z,9Z,12Z,15Z)) | 6543.53±614.65 | 7672.04±403.29** | ↑ |
| Flavonoids | 7-Prenyloxy-3',4'- dimethoxyisoflavone | 16.94±5.15 | 6.94±1.36** | ↓ |
|  | Malvidin 3-rutinoside | 0.00±0.00 | 40.35±6.96** | ↑ |
|  | Pelargonidin 3- (6''-malonylglucoside)-5-glucoside | 25.5±8.42 | 46.16±8.99** | ↑ |
|  | (+)-Myristinin A | 0.16±0.03 | 0.37±0.10* | ↑ |
| Sterols | Diginatin | 8.01±1.77 | 1.72±0.10** | ↓ |
|  | 1beta,3beta,5alpha,6beta-tetrahydroxyandrostan-17-one | 2.8±0.90 | 0.52±0.16** | ↓ |
|  | Hippuristanolide | 0.00±0.00 | 13.97±6.84** | ↑ |
| Moss | Cucurbitacin C | 2.16±0.79 | 4568.85±1192.68** | ↑ |
|  | Cucurbitacin O | 5.24±1.19 | 312.16±54.46** | ↑ |
|  | Cucurbitacin S | 17.96±4.28 | 2018.90±211.83** | ↑ |
| Fatty Acyls | 9-tetradecynoic acid | 6.17±0.62 | 0.67±0.20** | ↓ |
|  | 26:4(11Z,14Z,17Z,20Z) | 8.77±0.48 | 11.43±0.89** | ↑ |
|  | Citric acid | 77.04±4.06 | 83.71±3.20* | ↑ |
| Glycerolipids | DG(16:0/18:3(9Z,12Z,15Z)/0:0) | 139.24±57.12 | 296.03±38.19** | ↑ |
|  | DG(20:5(5Z,8Z,11Z,14Z,17Z)/0:0/20:5(5Z,8Z,11Z,14Z,17Z)) (d5) | 8.42±1.59 | 11.90±1.60* | ↑ |
| Other compounds | D-Maltose | 233.83±52.00 | 108.45±22.96** | ↑ |
|  | Uridine 5'-monophosphate | 350.28±163.06 | 788.49±191.08** | ↑ |

Asterisks indicated significant differences (Student t-test: *, P < 0.05; **, P < 0.01) relative to the grafted plants with muskmelon rootstock. “↑” and “↓” indicated that the fruit metabolite was up- and down-regulated in grafted plants with pumpkin rootstock compared to grafted plants with muskmelon rootstock. Numbers in third and fourth column represent average ± SD.
