# Supplementary material for: Metabolomic analysis of the occurrence of bitter fruits on grafted oriental melon plants
Source: PLoS One. 2019 Oct 10;14(10):e0223707. doi: 10.1371/journal.pone.0223707 (PMC6786619; doi:10.1371/journal.pone.0223707)
Supplement: Attachment — Submitted filename: PONE-D-19-19662_reviewer.pdf [file pone.0223707.s004.pdf]

PLOS ONE

# Metabolomic analysis for the occurrence of bitter fruits of grafting oriental melon plants

--Manuscript Draft--

| | |
|---|---|
| **Manuscript Number:** | PONE-D-19-19662 |
| **Article Type:** | Research Article |
| **Full Title:** | Metabolomic analysis for the occurrence of bitter fruits of grafting oriental melon plants |
| **Short Title:** | The cause of bitterness produced after grafting |
| **Corresponding Author:** | Wensheng Zhao<br>Hebei Agricultural University<br>Baoding, CHINA |
| **Keywords:** | |
| **Abstract:** | Grafting has been widely applied to melon (Cucumis melo L.) production to alleviate continuous cropping obstacles and control soil-borne diseases. However, grafting easily led to fruit quality decline. For example, bitter fruits sometimes occurred on grafted plants. But the physiological mechanism was still unclear. In this study, effects of different rootstocks on the taste of fruits of Balengcui, an oriental melon cultivar, during summer production were investigated. The results showed that all the grafted plants with Cucurbita maxima Duch. rootstocks produced bitter fruits, while the non-grafted plants and the grafted plants with muskmelon rootstocks produced no bitter fruit. Liquid Chromatography–Mass Spectrometry (LC-MS) and metabonomic analysis were performed to investigate the mechanism of the occurrence of bitter fruits. Metabolites comparisons of the fruits from grafted plants with Ribenxuesong as rootstock both with the non-grafted plants and grafted plants with muskmelon rootstock showed that 17 metabolites including phospholipids, cucurbitacins and flavonoids exhibited same changes. Three Cucurbitacins, Cucurbitacin O, Cucurbitacin C and Cucurbitacin S, increased dramatically. Ten phospholipids (PS(18:1(9Z)/18:2(9Z,12Z)), PS(P-18:0/15:0), PA(18:1(11Z)/18:1(11Z)), PE(16:0/18:0), PS(O-16:0/17:2(9Z,12Z)), PI(16:0/18:2(9Z,12Z)), PA (15:0/22:6(4Z,7Z,10Z,13Z,16Z,19Z)), PS(P-16:0/17:2(9Z,12Z)), PS(22:0/22:1(11Z)), PA(17:1(9Z) /0:0)) significantly decreased, while 2 PA (16:0/18:2 (9Z, 12Z) and 16:0/18:1 (11Z)), 2 flavonoids (Pelargonidin 3-(6''-malonylglucoside)-5-glucoside and Malvidin 3-rutinoside) significantly increased in fruits of grafted plants with Cucurbita maxima Duch. rootstocks. These metabolites involved the glycerophospholipid metabolic pathway, the mevalonate pathway and the phenylpropanoid pathway. |
| **Order of Authors:** | Wensheng Zhao |
| | Shuangshuang Zhang |
| | Lanchun Nie |
| | Qiang Cui |
| | Jiahao Wang |
| | Yaqian Duan |
| | Chang Ge |
| **Additional Information:** | |
| **Question** | **Response** |
| **Financial Disclosure**<br><br>Enter a financial disclosure statement that describes the sources of funding for the work included in this submission. Review the submission guidelines for detailed requirements. View published research | The authors are thankful for the financial support from the earmarked fund for Hebei Vegetables Innovation Team of Modern Agro-industry Technology Research System [HBCT2018030210].The funders had no role in study design, data collection and analysis, decision to publish, or preparation of the manuscript. |

Powered by Editorial Manager® and ProduXion Manager® from Aries Systems Corporation

| | |
|---|---|
| articles from *PLOS ONE* for specific examples.<br><br>This statement is required for submission and **will appear in the published article** if the submission is accepted. Please make sure it is accurate.<br><br>**Unfunded studies**<br>Enter: *The author(s) received no specific funding for this work.*<br><br>**Funded studies**<br>Enter a statement with the following details:<br>• Initials of the authors who received each award<br>• Grant numbers awarded to each author<br>• The full name of each funder<br>• URL of each funder website<br>• Did the sponsors or funders play any role in the study design, data collection and analysis, decision to publish, or preparation of the manuscript?<br>• **NO** - Include this sentence at the end of your statement: *The funders had no role in study design, data collection and analysis, decision to publish, or preparation of the manuscript.*<br>• **YES** - Specify the role(s) played.<br><br>* typeset | |
| **Competing Interests**<br><br>Use the instructions below to enter a competing interest statement for this submission. On behalf of all authors, disclose any competing interests that could be perceived to bias this work—acknowledging all financial support and any other relevant financial or non-financial competing interests.<br><br>This statement **will appear in the published article** if the submission is accepted. Please make sure it is accurate. View published research articles from *PLOS ONE* for specific examples. | The authors have declared that no competing interests exist. |

Powered by Editorial Manager® and ProduXion Manager® from Aries Systems Corporation

| | |
|---|---|
| **NO authors have competing interests**<br><br>Enter: *The authors have declared that no competing interests exist.*<br><br>**Authors with competing interests**<br><br>Enter competing interest details beginning with this statement:<br><br>*I have read the journal's policy and the authors of this manuscript have the following competing interests: [insert competing interests here]*<br><br>* typeset | |
| **Ethics Statement**<br><br>Enter an ethics statement for this submission. This statement is required if the study involved:<br><br>• Human participants<br>• Human specimens or tissue<br>• Vertebrate animals or cephalopods<br>• Vertebrate embryos or tissues<br>• Field research<br><br>Write "N/A" if the submission does not require an ethics statement.<br><br>General guidance is provided below. Consult the submission guidelines for detailed instructions. **Make sure that all information entered here is included in the Methods section of the manuscript.** | N/A |

Powered by Editorial Manager® and ProduXion Manager® from Aries Systems Corporation

| | |
|---|---|
| **Format for specific study types**<br><br>**Human Subject Research (involving human participants and/or tissue)**<br>• Give the name of the institutional review board or ethics committee that approved the study<br>• Include the approval number and/or a statement indicating approval of this research<br>• Indicate the form of consent obtained (written/oral) or the reason that consent was not obtained (e.g. the data were analyzed anonymously)<br><br>**Animal Research (involving vertebrate animals, embryos or tissues)**<br>• Provide the name of the Institutional Animal Care and Use Committee (IACUC) or other relevant ethics board that reviewed the study protocol, and indicate whether they approved this research or granted a formal waiver of ethical approval<br>• Include an approval number if one was obtained<br>• If the study involved *non-human primates*, add *additional details* about animal welfare and steps taken to ameliorate suffering<br>• If anesthesia, euthanasia, or any kind of animal sacrifice is part of the study, include briefly which substances and/or methods were applied<br><br>**Field Research**<br><br>Include the following details if this study involves the collection of plant, animal, or other materials from a natural setting:<br>• Field permit number<br>• Name of the institution or relevant body that granted permission | |
| **Data Availability**<br><br>Authors are required to make all data underlying the findings described fully available, without restriction, and from the time of publication. PLOS allows rare exceptions to address legal and ethical concerns. See the PLOS Data Policy and FAQ for detailed information. | Yes - all data are fully available without restriction |

| | |
|---|---|
| A Data Availability Statement describing where the data can be found is required at submission. Your answers to this question constitute the Data Availability Statement and **will be published in the article**, if accepted.<br><br>**Important:** Stating 'data available on request from the author' is not sufficient. If your data are only available upon request, select 'No' for the first question and explain your exceptional situation in the text box.<br><br>Do the authors confirm that all data underlying the findings described in their manuscript are fully available without restriction? | |
| **Describe where the data may be found in full sentences. If you are copying our sample text, replace any instances of XXX with the appropriate details.**<br><br>• If the data are **held or will be held in a public repository**, include URLs, accession numbers or DOIs. If this information will only be available after acceptance, indicate this by ticking the box below. For example: *All XXX files are available from the XXX database (accession number(s) XXX, XXX.).*<br>• If the data are all contained **within the manuscript and/or Supporting Information files**, enter the following: *All relevant data are within the manuscript and its Supporting Information files.*<br>• If neither of these applies but you are able to provide **details of access elsewhere**, with or without limitations, please do so. For example:<br><br>*Data cannot be shared publicly because of [XXX]. Data are available from the XXX Institutional Data Access / Ethics Committee (contact via XXX) for researchers who meet the criteria for access to confidential data.*<br><br>*The data underlying the results presented in the study are available from (include the name of the third party* | All relevant data are within the manuscript and its Supporting Information files. |

| | |
|---|---|
| *and contact information or URL).*<br>• This text is appropriate if the data are owned by a third party and authors do not have permission to share the data.<br><br>* typeset | |
| Additional data availability information: | |

Powered by Editorial Manager® and ProduXion Manager® from Aries Systems Corporation

Manuscript Click here to download Manuscript Manuscript.doc

# Metabolomic analysis for the occurrence of bitter fruits of grafting oriental melon plants

Shuangshuang Zhang, Lanchun Nie*, Wensheng Zhao*, Qiang Cui, Jiahao Wang, Yaqian Duan and Chang Ge

College of Horticulture, Hebei Agricultural University/Hebei Key Laboratory of Vegetable Germplasm Innovation and Utilization/Collaborative Innovation Center of Vegetable Industry of Hebei Province, Hebei, China

* Co-corresponding authors:

Lanchun Nie: 13784960296@139.com;

Wensheng Zhao: zhaowensheng@hebau.edu.cn.

**Short title:** The cause of bitterness produced after grafting

**Abstract**

Grafting has been widely applied to melon *(Cucumis melo* L.*)* production to alleviate continuous cropping obstacles and control soil-borne diseases. However, grafting easily led to fruit quality decline. For example, bitter fruits sometimes occurred on grafted plants. But the physiological mechanism was still unclear. In this study, effects of different rootstocks on the taste of fruits of Balengcui, an oriental melon cultivar, during summer production were investigated. The results showed that all the grafted plants with *Cucurbita maxima* Duch. rootstocks produced bitter fruits, while the non-grafted plants and the grafted plants with muskmelon rootstocks produced no bitter fruit. Liquid Chromatography–Mass Spectrometry (LC-MS) and metabonomic analysis were performed to investigate the mechanism of the occurrence of bitter fruits. Metabolites comparisons of the fruits from grafted plants with Ribenxuesong as rootstock both with the non-grafted plants and grafted plants with muskmelon rootstock showed that 17 metabolites including phospholipids, cucurbitacins and flavonoids exhibited same changes. Three Cucurbitacins, Cucurbitacin O, Cucurbitacin C and Cucurbitacin S, increased dramatically. Ten phospholipids (PS(18:1(9Z)/18:2(9Z,12Z)), PS(P-18:0/15:0), PA(18:1(11Z)/18:1(11Z)), PE(16:0/18:0), PS(O-16:0/17:2(9Z,12Z)), PI(16:0/18:2(9Z,12Z)), PA (15:0/22:6(4Z,7Z,10Z,13Z,16Z,19Z)), PS(P-16:0/17:2(9Z,12Z)), PS(22:0/22:1(11Z)), PA(17:1(9Z) /0:0)) significantly decreased, while 2 PA (16:0/18:2 (9Z, 12Z) and 16:0/18:1 (11Z)), 2 flavonoids (Pelargonidin 3-(6"-malonylglucoside)-5-glucoside and Malvidin 3-rutinoside)

significantly increased in fruits of grafted plants with *Cucurbita maxima* Duch. rootstocks. These metabolites involved the glycerophospholipid metabolic pathway, the mevalonate pathway and the phenylpropanoid pathway.

**Keywords:** melon, LC-MS, metabonomic analysis, bitterness, grafting, rootstocks

## Introduction

Melon (*Cucumis melo* L.) is an important horticultural crop [1]. In 2017, the planting area and yield were 490,327 hectares and 17,147,817t in the world according to Food and Agriculture Organization of the United Nations (FAO) [2]. Melon production played an important role in horticultural planting [3,4,5]. However, intensive production had led to continuous cropping obstacles and soil-borne diseases aggravation [6]. To alleviate continuous cropping obstacles and control soil-borne diseases, grafting has been widely applied to melon production [7]. However, grafting sometimes led to decline of fruit quality. Balengcui, an oriental melon cultivar in China known for its crispy fruit, was commonly grafted on the *Cucurbita maxima* Duch.. But the grafted plants produced bitter fruit during summer cultivation.

Cucurbitacins were the main compounds causing the bitterness of *Cucurbitaceae* fruits[8]. There were highly oxidized tetracyclic triterpenoid compounds synthesized by the methylovalerate pathway from acetyl CoA under the catalysis of series of enzymes [9]. Generally cultivated *Cucurbitaceae* plants do not produce bitter fruits. But some factors, such as low or high temperature, excessive nitrogen, grafting, plant growth regulators, and changes in the physical and chemical properties of soil may lead to coordination operation of some genes and induce synthesis of cucurbitacins [10-12]. Kano and Goto reported that during cucumber growth, plants with twice the amount of nitrogen fertilizer had a higher probability of bitter leaves and fruits than those with normal amount of nitrogen fertilizer [13]. The excessive nitrogen fertilizer lead to imbalance of N metabolism in cucumber plants, and the activity of HMG-CoA

reductase increased and resulted in the synthesis of cucurbitacin in cucumber plants. However, there were few reports on the mechanism about how grafting caused bitterness in fruits.

Internal and external factors cause coordinated physiological response. Metabolomics as a well-established method for characterizing the metabolism had been widely used to study the effects of environmental factors on plant physiological metabolism [14,15] and reveal the response mechanism of plants to biotic or abiotic stresses[16]. Meng Jie compared the metabolite profiles of high-flavonoids mutant and common Ginkgo leaves, and found 72 different metabolites related to the biosynthesis of flavonoids [17]. Metabonomics analysis revealed that phenylpropanoid biosynthesis pathway and lipid metabolism pathway regulated Ginkgo flavone biosynthesis. In this study, the effect of different rootstocks on the occurrence of bitter fruit of Balengcui during summer production was investigated. Liquid Chromatography–Mass Spectrometry (LC-MS) and metabonomic analysis were performed to investigate the mechanism of the occurrence of bitter fruits of the grafted plants.

## Materials and methods

### Plant materials and growth conditions

In order to investigate the effect of different rootstocks on the occurrence of bitter fruits, Seedlings of Balengcui with two cotyledons were grafted onto 6 *Cucurbita maxima* Duch. rootstocks ( Yemuyixiong, Ribenxuesong, Qingshengzhenjia, Jingxinzhen3, Feichangfuzuo and Jingyutianzhen1) and 2 *Cucumis melo* L. rootstocks

(Inbred line1 and Inbred line2). The detailed information about the rootstocks was shown in Table S1. The grafted and non-grafted seedlings with 3-4 euphylla were planted in the plastic house on June 20, 2018. Randomized block design with three replications was used. The area of each plot was 10m$^2$ and the planting space was 70*35cm. The temperature was 35°C /22°C of day/night and the relative air humidity was70~80%.

**Evaluation of bitter fruits**

During the flowering and fruit setting period, the fruits that flowered on the same day were marked at 10-12 nodes of the main vine. At fruit marketable mature stage, 25 marked fruits with the same maturity were taken from each plot. Twenty tasters were selected to taste all the fruits. They rinsed their mouth after each tasting to avoid affecting judgment to the next sample [18]. The fruit was defined as the bitter fruit when more than 10 peoples tasted bitter. The bitter score was evaluated with 10 points system and the average value was used.

**Liquid Chromatography–Mass Spectrometry (LC-MS) analysis**

The bitter fruits with the highest bitter score from grafted plants with Ribenxuesong as rootstock, non-bitter fruits from grafted plants with muskmelon Inbred Line 1 as rootstock and non-bitter fruit from non-grafted plants were taken to metabolomic analysis. Fruit samples (60 mg) with 20μl internal standard solutions (L-2-chloro-phenylalanine, 0.3 mg/mL) and 1ml methanol were broken into homogenate and proceeded ultrasonic extraction for 45 min. Centrifugation and the supernatant were taken to the LC-MS analysis.

LC-MS was performed by a Waters UPLC I-class system which equipped with a binary solvent delivery manager and a sample manager, and a Waters VION IMS Q-TOF Mass Spectrometer with an electrospray ionization (Waters，Milford, USA) which can operate in positive or negative ion mode. Acquity BEH C18 column (100 mm × 2.1 mm i.d., 1.7 μm) was used as chromatographic column and the temperature was maintained at 45 °C. Mobile phase A and B were water and acetonitrile both including 0.1% formic acid, respectively. The flow rate was 0.40 ml/min and the injection volume was 3.00μl. The gradient elution program was listed as followed: 5–20 % B, 0–2 min, 20–60 % B, 2–8 min, 60–100 % B, 8–12 min, 100 % B , 2 min, 100 % to 5 % B, 14–14.5 min, 5 % B, 1 min. The temperature of ion source and desolvation were 120℃ and 500℃, respectively. The desolvation gas flow rate is 900 L/h. Mass spectrum scan ranged from 50 to 1,000 m/z and the scanning time was 0.1 s with the interval of 0.02s.

## Data analysis

Progenesis QI (Waters Corporation, Milford, USA) was used to perform baseline filtration, peak identification, search characterization, integration, retention time correction, peak alignment and normalization. The secondary fragments from metabolite detection were matched to the standard position in database to get the data matrix consist of retention time, mass-to-charge ratio (or metabolite name), and peak intensity. SIMCA-14.1 (Umetrics AB, Umea, Sweden) was used to implement PCA and OPLS-DA multivariate statistical analysis. The differential metabolites were initially screened based on the VIP (Variable Importance in the Projection) >1.

Student t-test was performed by SPSS11.5 and significantly different metabolites were screened by $P < 0.05$.

## Results

### Effects of different rootstocks on the occurrence of bitter fruits

The results showed that all the grafted plants with *Cucurbita maxima* Duch. rootstocks produced bitter fruits with different proportions (Table 1).The highest proportion of bitter fruits was 72% in the grafted plants with Ribenxuesong as rootstock. However, no bitter fruit was found in all grafted plants with muskmelon rootstocks (Inbred line 1 and 2) and all non-grafted plants. These data indicated that rootstocks caused the occurrence of bitter fruits of Balengcui grafted plants during summer cultivation.

**Table 1 Evaluation of bitter fruits**

| Rootstocks | Number of bitter fruits | Total number of fruits | Rate of bitter fruit (%) |
|---|---|---|---|
| Yemuyixiong | 7 | 25 | 28 |
| Ribenxuesong | 18 | 25 | 72 |
| Qingshengzhenjia | 10 | 25 | 40 |
| Jingxinzhen3 | 12 | 25 | 48 |
| Feichangfuzuo | 10 | 25 | 40 |
| Jingyutianzhen1 | 13 | 25 | 52 |
| Muskmelon Inbred line1 | 0 | 25 | 0 |
| Muskmelon Inbred line2 | 0 | 25 | 0 |
| Non-grafted | 0 | 25 | 0 |

### Identification and analysis of fruits metabolites

LC-MS ion flow maps of bitter fruits from grafted plants with Ribenxuesong as rootstock, non-bitter fruits from grafted plants with muskmelon Inbred Line 1 as rootstock and non-bitter fruit from non-grafted plants were shown in Fig1. The number and intensity of metabolites in fruits of grafted plants with Ribenxuesong as

rootstock had significant difference compared with the fruits of non-grafted plants and grafted plants with muskmelon rootstock in positive and negative ion mode (Fig 1A and 1B). By progenesis QI software analysis and identification, 1194 metabolites were identified (Fig 2), of which 27% were fatty acyl metabolites, 23% were glycerol phospholipids metabolites, 12% were sterol lipid metabolites, 11% were polyketide glycosides metabolites, 6% were glycerol metabolites, 5% were prenol lipid metabolites, 4% were sphingolipids metabolites, 2% were steroid and derivative metabolites, carboxylic acid and derivative metabolites and organic oxide metabolites. There were also 1% amino acids and 5% other compounds.

**Fig 1. Total ion current maps of melon fruits from non-grafted plants and grafted plants with muskmelon or pumpkin rootstocks.** (A) Total ion current maps in positive ion mode. (B) Total ion current maps in negative ion mode.

**Fig 2. Classification of identified fruit metabolites.** Different classifications of fruit metabolites were shown as different colors. Black, fatty acyls. Blue, glycerophospholipids. Orange, sterol lipids. Gray, polyketides. Green, glycerolipids. Purple, prenol lipids. Red, sphingolipids. Light blue, steroids and steroid derivatives. Yellow, carboxylicacids and derivatives. Dark red, organooxygen compounds. Pink, amino acids. Light green, other metabolites.

The principal component analysis (PCA) was performed to verify the data and the PCA score map was shown in Fig 3 ($R^2X$=0.763, $Q^2$=0.219). All the samples were

uniformly distributed in T2 ellipse which indicated that there were no abnormal sample points. Sample points of the same group were obviously clustered together and sample points from three kinds of fruit sample can be completely distinguished in the overall distribution. These results indicated that there were significant differences in metabolites among fruits from non-grafted plants and grafted plants with Ribenxuesong or muskmelon Inbred Line 1 as rootstocks. The model parameter was $R^2X$=0.763, which fits well and can be used for screening and analysis of differential metabolites.

**Fig 3. PCA score plot of melon fruits from non-grafted plants and grafted plants with muskmelon or pumpkin rootstocks.** $R^2X$=0.763, $Q^2$=0.219.

In order to further analyze the fruits metabolites, the orthogonal partial least-squares discrimination analysis (OPLS-DA) models were established. The score maps were shown in Fig 4. The OPLS-DA model parameters of fruits from grafted plants with Ribenxuesong as rootstock and non-grafted plants were $R^2X$=0.586, $R^2Y$=0.955, $Q^2$=0.873 (Fig 4A), and the parameters of fruits from grafted plants with Ribenxuesong as rootstock and with muskmelon Inbred Line 1 as rootstock were $R^2X$=0.598, $R^2Y$=0.972, $Q^2$=0.881 (Fig 4B). The parameters of $R^2$ and $Q^2$ were all more than 0.5. Six replicates of each group were closely concentrated and the sample points of different groups were well separated. Therefore, the models were used to screen different metabolites.

**Fig 4. OPLS-DA score plot of melon fruits from non-grafted plants and grafted plants with muskmelon or pumpkin rootstocks.** (A) OPLS-DA score plot of melon fruits from non-grafted plants and grafted plants with pumpkin rootstocks. $R^2X=0.586$, $R^2Y=0.955$, $Q^2=0.873$. (B) OPLS-DA score plot of melon fruits from grafted plants with muskmelon rootstocks and grafted plants with pumpkin rootstocks. $R^2X=0.598$, $R^2Y=0.972$, $Q^2=0.881$.

## Effects of different rootstocks on fruits metabolites

According to the results of OPLS-DA with the screening condition of VIP>1 and $P<0.05$, 37 metabolites including phospholipids, sterols, flavonoids, terpenoids significantly changed in bitter fruits of grafted plants with Ribenxuesong as rootstock compared with fruits of non-grafted plants (Table S2). There were 16 phospholipids significantly decreased and 6 significantly increased. For sterol metabolites, 4 significantly increased and 2 significantly decreased. There were 3 flavonoids metabolites significantly increased and 1 significantly decreased. The terpenoids metabolites, cucurbitacin O, cucurbitacin C and cucurbitacin S increased 398.53, 203.33 and 238.21 times, respectively. Another 2 metabolites, spiramycin and monoisobutyl phthalic acid also significantly increased in fruits of grafted plants with Ribenxuesong as rootstock.

Compared metabolites of bitter fruits of the grafted plants with Ribenxuesong as rootstock with the fruits of the grafted plants with muskmelon Inbred Line 1 as rootstock, 33 differential metabolites including 16 phospholipids, 4 flavones, 3 sterols,

3 terpenoids, 3 organic acids and 4 other compounds significantly changed (Table S3). Among 16 phospholipid metabolites, PA (16:0/18:2 (9Z, 12Z), PA (16:0/18:1 (11Z), PA (18:1 (9Z)/18:4 (6Z, 9Z, 12Z, 15Z) increased, while the other 13 glycerophospholipids decreased significantly. Among 4 flavonoids, Pelargonidin 3-(6''-malonylglucoside)-5-glucoside, Malvidin 3-rutinoside and (+)-Myristinin A increased, while 7-Prenyloxy-3',4'-dimethoxyisoflavone decreased. For 3 sterols, hippuristanolide increased, while 1β, 3β, 5α, 6β-tetrahydroxyandrostan-17-one and diginatin decreased. Three terpenoids, Cucurbitacin O、Cucurbitacin C and Cucurbitacin S, showed 89.36 times, 337.81 times and 168.58 times increase, respectively. 9-tetradecynoic acid decreased. Citric acid, 26:4(11Z,14Z,17Z,20Z) and glycerol esters, DG (16:0/18:3 (9Z, 12Z, 15Z)/0:0) and DG (20:5 (5Z, 8Z, 11Z, 14Z, 17Z)/0:0/20:5 (5Z, 8Z, 11Z, 14Z, 17Z)) and other 2 metabolites, D-Maltose and Uridine 5'-monophosphate increased.

Overall, compared together with the non-bitter fruits produced by non-grafted plants and grafted plants with muskmelon Inbred Line 1 rootstock, 17 metabolites including 3 terpenoids, 2 flavonoids and 12 phospholipids in bitter fruits of grafted plants with Ribenxuesong as rootstock showed same change (Fig 5). The phospholipids, (PS(18:1(9Z)/18:2(9Z,12Z)), PS(P-18:0/15:0), PA(18:1(11Z)/18:1(11Z)), PE(16:0/18:0), PS(O-16:0/17:2(9Z,12Z)), PI(16:0/18:2(9Z,12Z)), PA (15:0/22:6(4Z,7Z,10Z,13Z,16Z,19Z)), PS(P-16:0/17:2(9Z,12Z)), PS(22:0/22:1(11Z)) and PA(17:1(9Z) /0:0)) were significantly decreased, while PA (16:0/18:2 (9Z, 12Z)) and PA (16:0/18:1 (11Z)

were significantly increased (Fig 5A). The 3 terpenoids metabolites, Cucurbitacin O, Cucurbitacin C and Cucurbitacin S, dramatically increased (Fig 5B). 2 Flavonoids, Pelargonidin 3-(6'-malonyl glucoside) -5-glucoside and Malvidin 3-rutinoside, were significantly increased (Fig 5C). These 17 differential metabolites were mapped to KEGG and Metabo Analyst 3.0 websites. Pathway enrichment analysis was carried out by combining -log P and Impact values. The involved metabolic pathways were glycerol phospholipid pathway, methylovalerate pathway and phenylpropane pathway.

**Fig 5. Significantly changed fruit metabolites identified from the comparison between grafted plants with pumpkin rootstock and non-grafted plants or grafted plants with muskmelon rootstock.** Seventeen significantly changed fruit metabolites were identified including phospholipids (A), cucurbitacin (B) and flavonoids (C). Number 1-12 represent PS(18:1(9Z)/18:2(9Z,12Z)), PS(P-18:0/15:0), PA(18:1(11Z)/18:1(11Z)), PE(16:0/18:0), PA(16:0/18:1(11Z)), PA(16:0/18:2(9Z,12Z)), PS(O-16:0/17:2(9Z,12Z)), PI(16:0/18:2(9Z,12Z)), PA (15:0/22:6(4Z,7Z,10Z,13Z,16Z,19Z)), PS(P-16:0/17:2(9Z,12Z)), PS(22:0/22:1(11Z)), PA(17:1(9Z) /0:0), respectively. Number 13-15 represent Cucurbitacin C, Cucurbitacin S, Cucurbitacin O, respectively. Number 16 and 17 represent Pelargonidin 3-(6"-malonylglucoside)-5-glucoside and Malvidin 3-rutinoside, respectively. The lowercase represents $P<0.05$ and the capital represents $P<0.01$. The letter before "/" indicates the significance between grafted plants with pumpkin

rootstock and non-grafted plants. The letter after "/" indicates the significance between grafted plants with pumpkin rootstock and muskmelon rootstock.

## Discussion

Bitterness often occurred in melon, cucumber, gourd and other cucurbitaceae plants and affects quality of fruits. Environmental and endogenous factors such as temperature, nutrition and plant hormones could induce the bitterness [11, 12]. In this study, the results showed that all the grafted plants with *Cucurbita maxima* Duch. rootstocks produced bitter fruits, but no bitter fruit was found on the non-grafted plants or plants with muskmelon rootstock. These suggested that pumpkin rootstock caused the bitterness of Balengcui fruit during summer cultivation.

LC–MS and metabonomic analysis methods were applied to investigate the mechanism for the occurrence of bitter fruits. Metabolites comparisons of the fruits from grafted plants with Ribenxuesong as rootstock both with the non-grafted plants and grafted plants with muskmelon rootstock showed that 17 metabolites exhibited same change (Fig 5). Three cucurbitacins increased dramatically in grafted plants with Ribenxuesong as rootstock. Cucurbitacins, as secondary metabolites of highly oxidized tetracyclic triterpenoids, were the main compounds caused bitterness in cucurbitaceous fruits, such as cucumber, pumpkin and squash [19-21]. But why the cucurbitacins increased dramatically in the fruit of grafted plants with Ribenxuesong as rootstock was not unclear. Wu *et al* reported that the grafted plants with *Cucurbita maxima* Duch. as rootstock exhibited lower activity of antioxidant enzymes and

higher level of reactive oxygen species (ROS) [22, 23]. The balance of active oxygen metabolism system was easily destroyed. The reactive oxygen destructed the cell membrane structure and physiology integrity [24, 25]. This study showed that most of the phospholipids in the fruits of grafted plants with Ribenxuesong as rootstock significantly decreased compared with those of non-grafted plants or grafted plants with muskmelon rootstock (Fig 5A). Phospholipids were important membrane components, which are essential for maintaining cell stability and protecting plants against stress [26]. *Cucurbita maxima* Duch. is less high-temperature-resistance than melon. We speculated that during summer cultivation, high temperature caused imbalance of reactive oxygen metabolism system in grafted plants with pumpkin rootstocks, which resulted in membrane lipid peroxidation and phospholipid decomposition into fatty acids [27]. Fatty acids were further oxidized to produce large amounts of acetyl coenzyme A, and Cucurbitacins were synthesized from acetyl CoA via methylovalerate pathway [28].

PAs were also important lipid signaling molecules found in plants and called the lipid second messenger which involved in mediating the production and response of peroxides and affected the accumulation of oxidized lipids [29-31]. Some PAs showed increase under the abiotic stress in plants [32]. In this study, the increase of PA (16:0/18:1 (11Z)) and PA (16:0/18:2 (9Z, 12Z)) in the fruit of grafted plant with Ribenxuesong as rootstocks indicated that membrane lipid peroxidation might occur in grafted plants under high temperature during summer cultivation (Fig 5A).

Flavonoids are secondary metabolites of phenols, which play an important role in plant response to environmental signals. Flavonoids, for example, can protect plant tissues from damage by high-energy wavelengths (ultraviolet-A and ultraviolet-B) under high temperature and strong light stress [33, 34]. In this study, 2 flavonoids (Pelargonidin 3-(6''-malonylglucoside)-5-glucoside and Malvidin 3-rutinoside) were significantly increased in fruit of grafted plants with Ribenxuesong as rootstock. The results suggested that the phenylpropane metabolism in Balengcui fruits of the grafted plants with Ribenxuesong as rootstocks was enhanced and more flavonoids were synthesized to resist high temperature during summer cultivation.

## Conclusions

All the grafted plants with *Cucurbita maxima* Duch. as rootstocks produced bitter fruits, but no bitter fruit was found on the non-grafted plants or plants with muskmelon rootstock. Metabolites comparisons of the fruits from grafted plants with Ribenxuesong as rootstock both with the non-grafted plants and grafted plants with muskmelon rootstock showed that 17 metabolites including phospholipids, cucurbitacins and flavonoids exhibited same change. Three Cucurbitacins, Cucurbitacin O, Cucurbitacin C and Cucurbitacin S, increased dramatically. Ten phospholipids significantly decreased, while PA (16:0/18:1 (11Z)), PA (16:0/18:2 (9Z, 12Z)) and 2 flavonoids(Pelargonidin 3-(6''-malonylglucoside)-5-glucoside and Malvidin 3-rutinoside) increased significantly in the fruit of grafted plant with Ribenxuesong as rootstocks. These 17 differential metabolites involved glycerol

phospholipid metabolic pathway, methylovalerate pathway and phenylpropane pathway.

## Supporting Information

**Table S1** Information of different grafting rootstocks

**Table S2** Comparison of metabolites in fruits of grafted plants with Ribenxuesong as rootstock and non-grafted plants

**Table S3** Comparison of metabolites in fruits of grafted plants with Ribenxuesong as rootstock and grafted plants with muskmelon as rootstock


## Acknowledgements

This study was supported by the earmarked fund for Hebei Vegetables Innovation Team of Modern Agro-industry Technology Research System [HBCT2018030210]. The authors are grateful to members of the lab for technical assistance and stimulating discussions.


## Author contributions

SZ, LN and WZ conceived and designed research. SZ conducted most of experiments and wrote the manuscript with LN and WZ. QC and JW analyzed the data. YD and CG helped for process of the whole experiment. All authors read and approved the manuscript.

## References:


1. Pitrat M. Melon genetic resources: phenotypic diversity and horticultural taxonomy. In: Grumet R, Katzir N, Garcia-Mas J, editors. Genetics and genomics of the Cucurbitaceae. New York: Springer Publishing Company; 2016. pp. 25–60.

2. Available from: http://www.fao.org/faostat/en/#data/QC. html Cited 4 April 2019.

3. Kim HA, Shin A, Lee M, Lee H, Lee H, Ahn J, et al. De novo transcriptome analysis of *Cucumis melo* L. var. makuwa. Molecules and Cells. 2016; 39(2): 141–148.

4. Mascarell-Creus A, Cañizares J, Vilarrasa-Blasi J, Mora-García S, Blanca J, Gonzalez-Ibeas D, et al. An oligo-based microarray offers novel transcriptomic approaches for the analysis of pathogen resistance and fruit quality traits in melon (*Cucumis melo* L.). BMC Genomics. 2009; 10(1): 467.

5. Ricardez-Salinas M, Huitron-Ramirez MV, Tello-Marquina JC, Camacho-Ferre F. Planting density for grafted melon as an alternative to methyl bromide use in Mexico. Scientia Horticulturae. 2010; 126(2): 236–241.

6. Aloni B, Cohen R, Karni L, Aktas H, Edelstein M. Hormonal signaling in rootstock–scion interactions. Scientia Horticulturae. 2010;127: 119–126.

7. Kumar P, Rana S, Sharma P, Negi V. Vegetable grafting: a boon to vegetable growers to combat biotic and abiotic stresses. Himachal Journal of Agricultural Research. 2015; 41(2): 1–5.

8. Rehm S, Enslin PR, Meeuse A, Wessels JH. Bitter principles of the Cucurbitaceae.

VII.—the distribution of bitter principles in this plant family. Journal of the Science of Food and Agriculture. 1957; 8: 679–686.

9. Zhou Y, Ma Y, Zeng J, Duan L, Xue X, Wang H, et al. Convergence and divergence of bitterness biosynthesis and regulation in Cucurbitaceae. Nature Plants. 2016; 2: 16183.

10. Pittenger DR. California master gardener handbook. California: University of California. Division of Agriculture and Natural Resources Publication; 2002.

11. Shang Y, Ma Y, Zhou Y, Zhang H, Duan L, Chen H, et al. Biosynthesis, regulation, and domestication of bitterness in cucumber. Science. 2014; 346(6213): 1084–1088.

12. Kano Y, Goto H, Fukuda H, Ishimoto K. Relationship between the occurrence of bitter fruit and nitrogen content, especially amino acid nitrogen and protein contents in the leaf and peel in cucumber (*Cucumis sativus* L., cv. *Kagafutokyuri*). Journal of the Japanese Society for Horticultural Science. 2001; 70: 438–442.

13. Kano Y, Goto H. Relationship between the occurrence of bitter fruit in cucumber (Cucumis sativus L.) and the contents of total nitrogen, amino acid nitrogen, protein and HMG-CoA reductase activity. Scientia Horticulturae. 2003; 98(1): 1–8.

14. Cramer GR, Ergül A, Grimplet J, Tillett RL, Tattersall EA, Bohlman MC, et al. Water and salinity stress in grapevines: early and late changes in transcript and metabolite profiles. Functional & Integrative Genomics. 2007; 7: 111–134.

15. Zuther E, Koehl K, Kopka J. Comparative metabolome analysis of the salt response in breeding cultivars of rice. In: Advances in molecular breeding toward

drought and salt tolerant crops. Dordrecht: Springer Publishing Company; 2007. pp. 285–315.

16. Zhao L, Huang Y, Hu J, Zhou H, Adeleye AS, Keller AA, et al. 1H NMR and GC-MS based metabolomics reveal defense and detoxification mechanism of cucumber plant under nano-Cu stress. Environmental Science & Technology. 2016; 50(4): 2000–2010.

17. Meng J, Wang B, He G, Wang Y, Tang X, Wang S, et al. Metabolomics integrated with transcriptomics reveals redirection of the phenylpropanoids Metabolic flux in *Ginkgo biloba.* Journal of Agricultural and Food Chemistry. 2019; 67(11): 3284–3291.

18. Horie H, Ito H, Ippoushi K, Azuma K, Sakata Y, Igarashi I. Cucurbitacin C—Bitter principle in cucumber plants. Japan Agricultural Research Quarterly: 2007; 41(1): 65–68.

19. Mukherjee PK, Nema NK, Maity N, Sarkar BK. Phytochemical and therapeutic potential of cucumber. Fitoterapia. 2013; 84: 227–236.

20. Rymal KS, Chambliss OL, Bond MD, Smith DA. Squash containing toxic cucurbitacin compounds occurring in California and Alabama. Journal of Food Protection. 1984; 47(4): 270–271.

21. Sun OC, Yong JK, Sang UP. An updated review of cucurbitacins and their biological and pharmacological activities. EXCLI Journal 2015; 14:562–566.

22. Wu Y, Chen Y, Zhao Y. Effect of pumpkin stocks on growth, development, yield and quality of drafted muskmelon. Fujian Journal of Agricultural Sciences. 2006;

21(4):354–369.

23. Wu Y, Zhao Y. The physiological influence on grafted muskmelon of different pumpkin stocks. Chinese Agricultural Science Bulletin. 2007; 23(11):253–256.

24. Catalá A. Lipid peroxidation of membrane phospholipids generates hydroxy-alkenals and oxidized phospholipids active in physiological and/or pathological conditions. Chemistry and Physics of Lipids. 2009; 157: 1–11.

25. Reis A, Spickett CM. Chemistry of phospholipid oxidation. Biochimica et Biophysica Acta (BBA)-Biomembranes. 2012; 1818: 2374–2387.

26. Meijer HJ, Munnik T. Phospholipid-based signaling in plants. Annual Review of Plant Biology. 2003; 54(1): 265–306.

27. Leshem YY. Membrane phospholipid catabolism and Ca2+ activity in control of senescence. Physiologia Plantarum. 1987; 69(3): 551–559.

28. Gershenzon J. Metabolic costs of terpenoid accumulation in higher plants. Journal of Chemical Ecology. 1994; 20(6): 1281–1328.

29. Jacob T, Ritchie S, Assmann SM, Gilroy S. Abscisic acid signal transduction in guard cells is mediated by phospholipase D activity. Proceedings of the National Academy of Sciences. 1999; 96(21): 12192–12197.

30. Katagiri T, Takahashi S, Shinozaki K. Involvement of a novel Arabidopsis phospholipase D, AtPLDδ, in dehydration- inducible accumulation of phosphatidic acid in stress signalling. The Plant Journal. 2001; 26(6): 595–605.

31. Li W, Wang R, Li M, Li L, Wang C, Welti R, et al. Differential degradation of extraplastidic and plastidic lipids during freezing and post-freezing recovery in

Arabidopsis thaliana. Journal of Biological Chemistry. 2008; 283(1): 461–468.

32. Drøbak BK, Watkins PA. Inositol (1, 4, 5) trisphosphate production in plant cells: an early response to salinity and hyperosmotic stress. FEBS Letters. 2000; 481(9): 240–244.

33. Samanta A, Das G, Das SK. Roles of flavonoids in plants. Carbon.2011;100(6): 12–35.

34. Mola J, Grotewold E, Koes R. How genes paint flowers and seeds. Trends in Plant Science. 1998; 3(6): 212-217.

Click here to download Figure Fig 1.tif

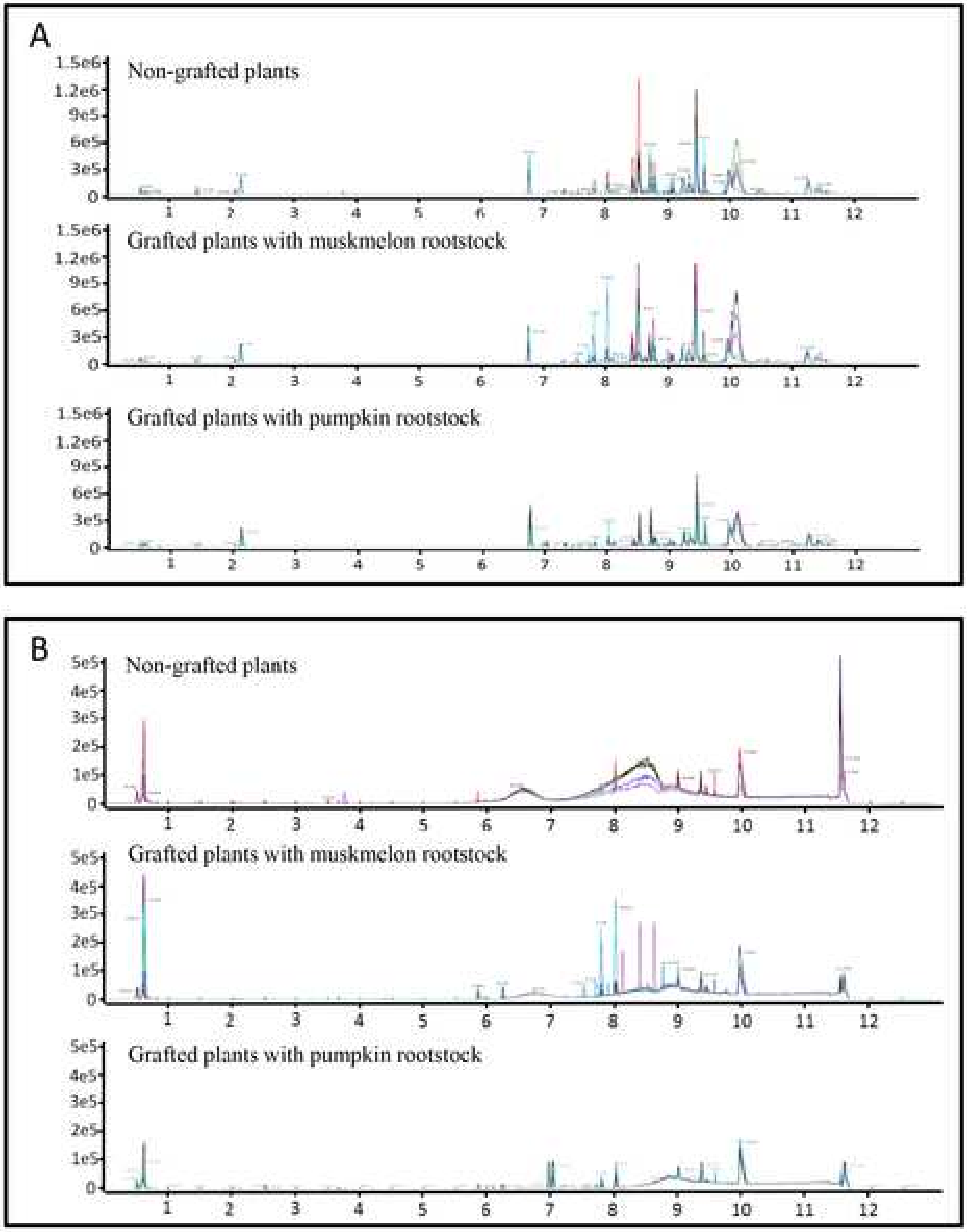

Click here to download Figure Fig 2.tif

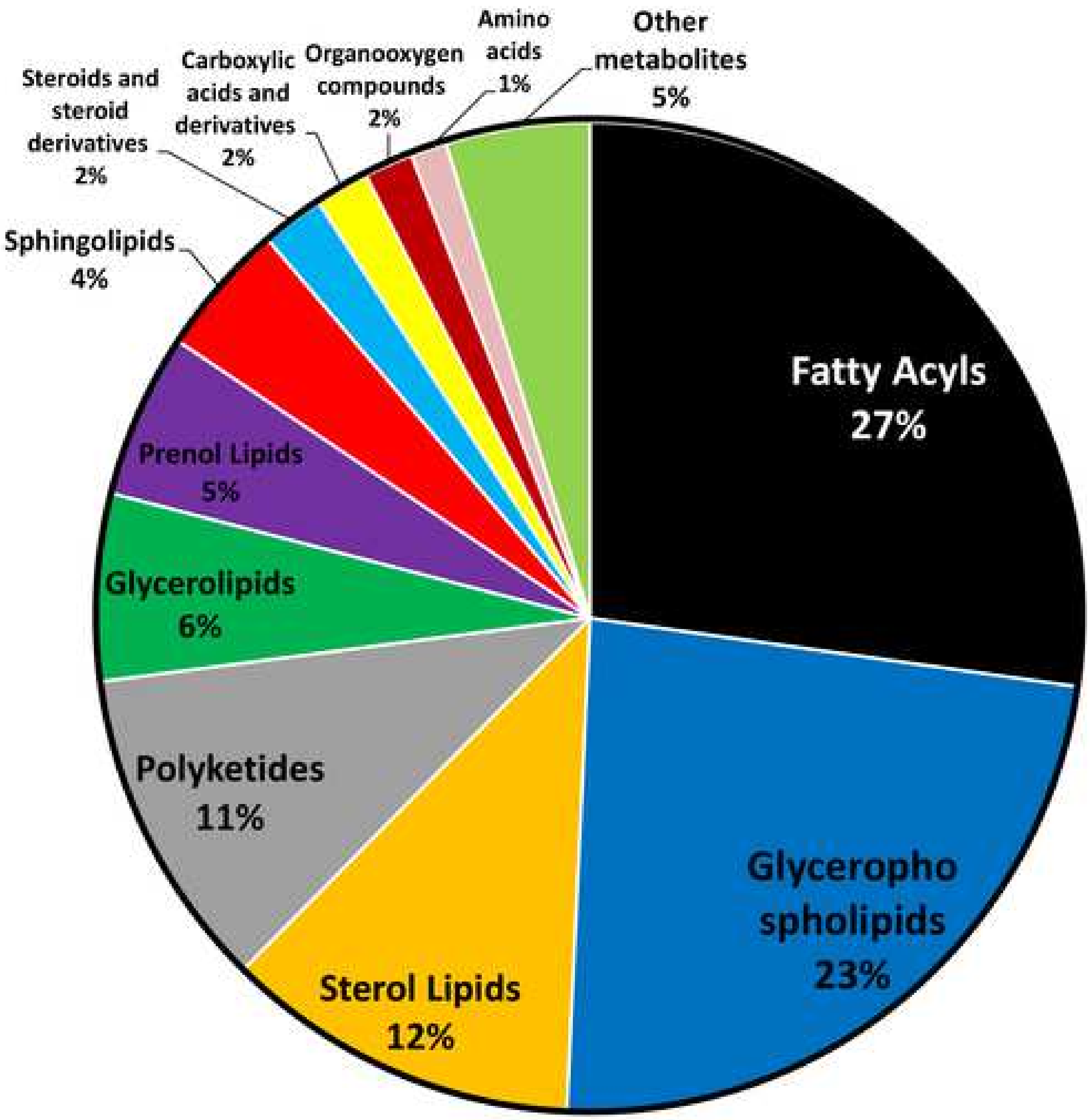

Click here to download Figure Fig 3.tif

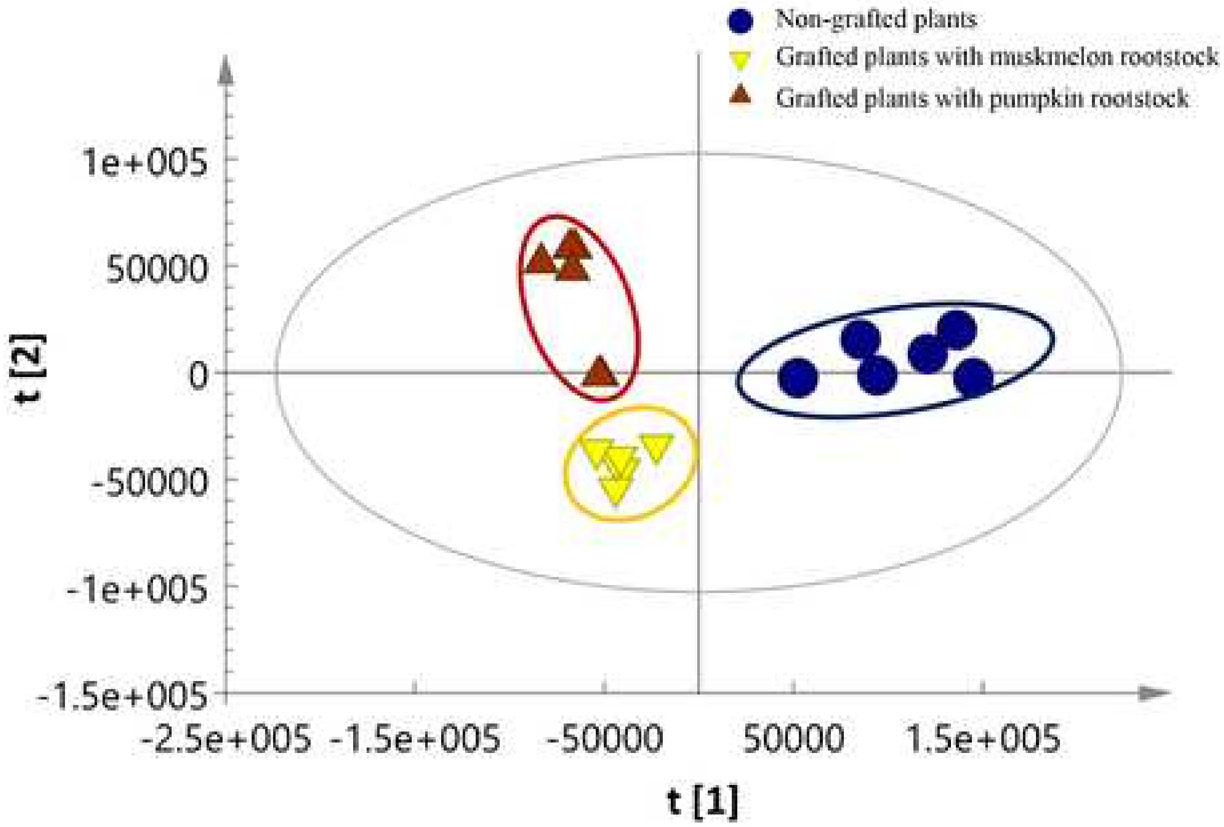

Click here to download Figure Fig 4.tif

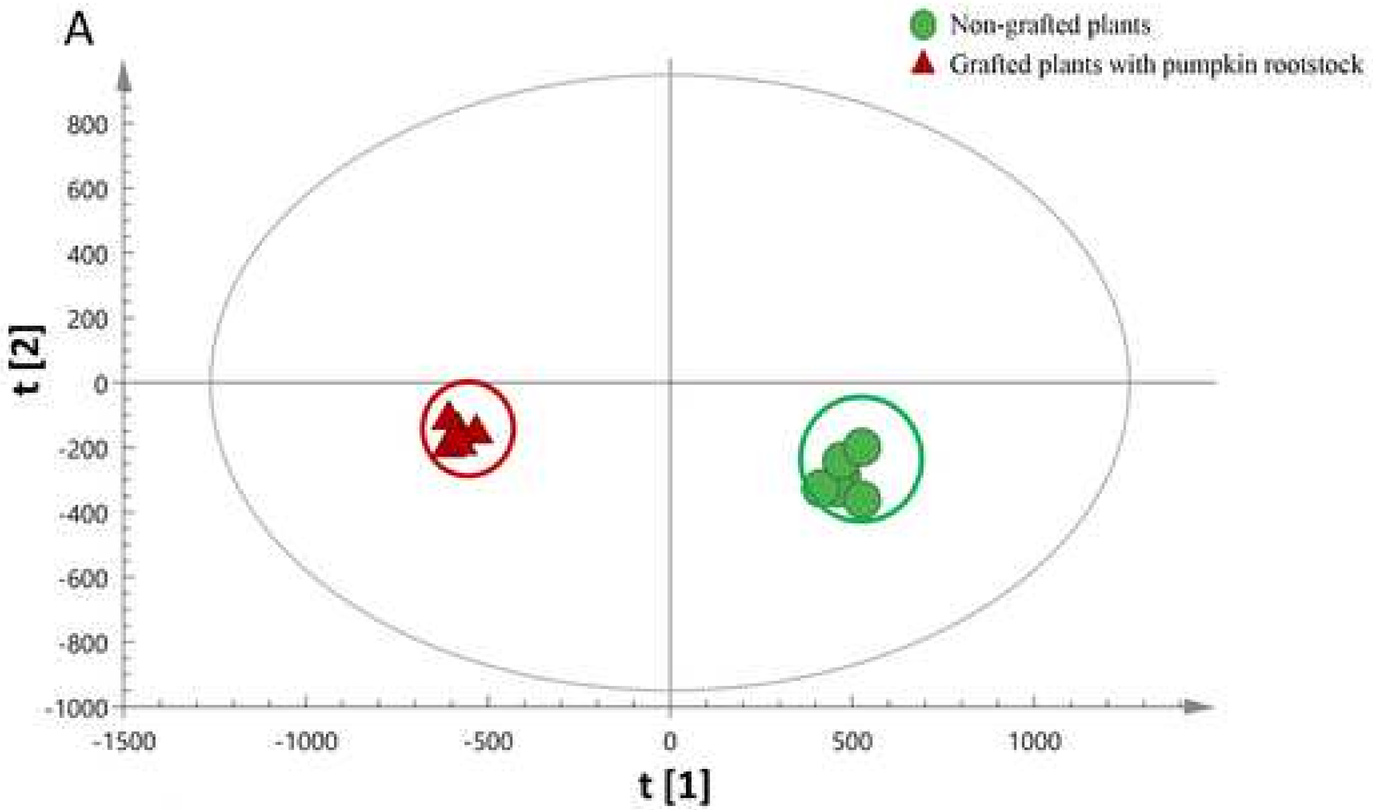


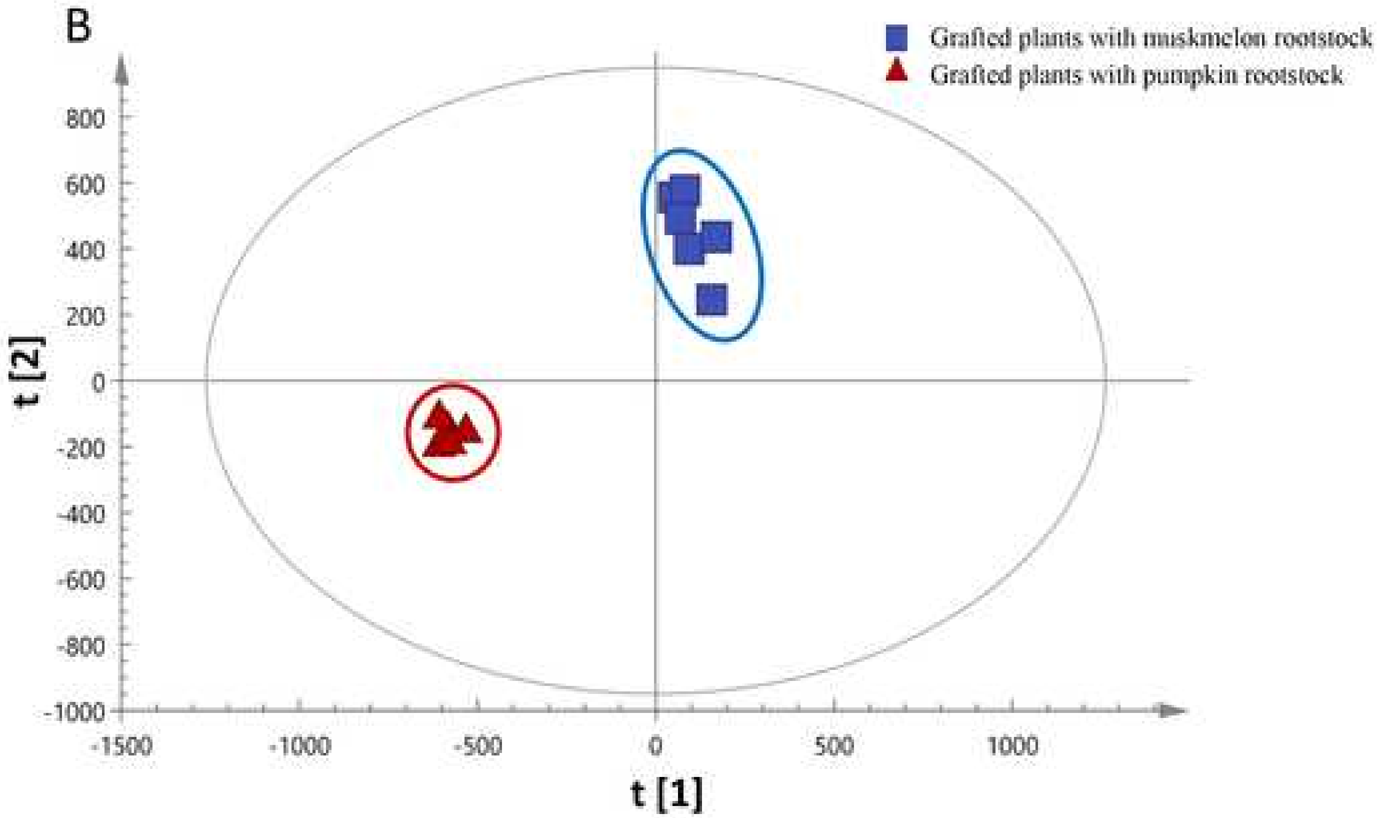

Click here to download Figure Fig 5.tif

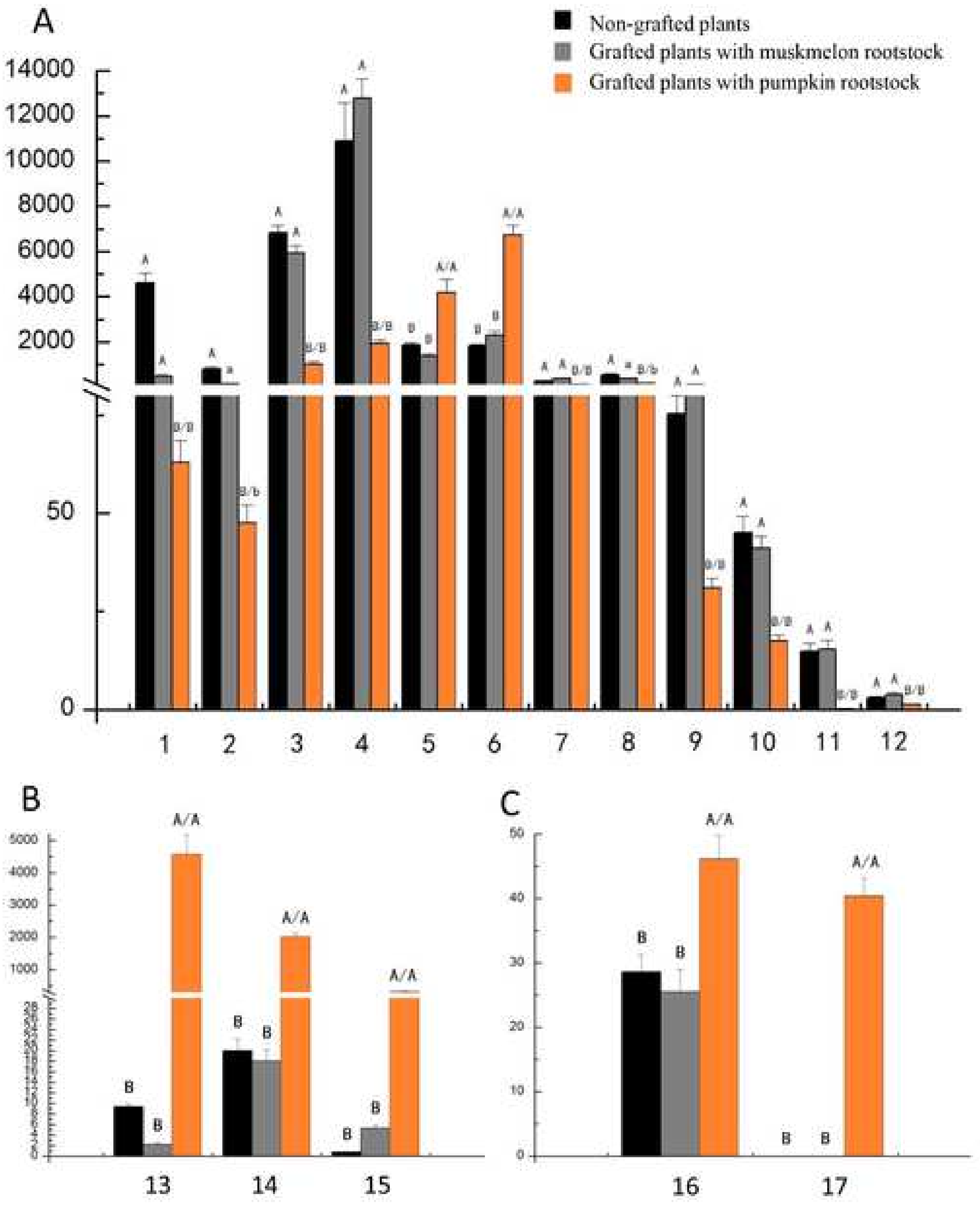

Click here to access/download
**Supporting Information**
Supporting Information.pdf
