# Supplementary material for: Metabolomic analysis of the occurrence of bitter fruits on grafted oriental melon plants
Source: PLoS One. 2019 Oct 10;14(10):e0223707. doi: 10.1371/journal.pone.0223707 (PMC6786619; doi:10.1371/journal.pone.0223707)
Supplement: Attachment — Submitted filename: Response to Reviewers.docx [file pone.0223707.s005.docx]

Dear editor,

We appreciate these comments and revised the manuscript as suggested by the academic editor and reviewer. We hope that the revised manuscript has fully addressed the comments so that it is now suitable for publication in *PLOS ONE*. Details were listed:

*Thank you for submitting your manuscript to PLOS ONE. After careful consideration, we feel that it has merit but does not fully meet PLOS ONE’s publication criteria as it currently stands. Therefore, we invite you to submit a revised version of the manuscript that addresses the points raised during the review process.*

**---Response**：We appreciate this comment. We have revised our manuscript and addressed each point raised during the review process.

*We would appreciate receiving your revised manuscript by Oct 14 2019 11:59PM. When you are ready to submit your revision, log on to https://www.editorialmanager.com/pone/ and select the 'Submissions Needing Revision' folder to locate your manuscript file.*
**---Response**：Thank you.

*If you would like to make changes to your* *financial disclosure, please include your updated statement in your cover letter.*
**---Response**：Thank you. No changes were made to our financial disclosure.

*To enhance the reproducibility of your results, we recommend that if applicable you deposit your laboratory protocols in protocols.io, where a protocol can be assigned its own identifier (DOI) such that it can be cited independently in the future. For instructions see:* [*http://journals.plos.org/plosone/s/submission-guidelines#loc-laboratory-protocols*](http://journals.plos.org/plosone/s/submission-guidelines#loc-laboratory-protocols)
**---Response**：We appreciate this comment. However, the methods used in this study were routine metabolomics analysis and there were no new approaches or insights. Therefore, we chose not deposit our laboratory protocols in protocols.io. If we develop new protocols in the future, we'd like to share it to protocols.io.

*Please include the following items when* *submitting your revised manuscript:*

- *A rebuttal letter that* *responds to each point raised by the academic editor and reviewer(s). This letter should be uploaded as separate file and labeled 'Response to Reviewers'.*
- *A marked-up copy of your manuscript that highlights changes made to the original version. This file should be uploaded as separate file and labeled 'Revised Manuscript with Track Changes'.*
- *An unmarked version of your revised paper without tracked changes. This file should be uploaded as separate file and labeled 'Manuscript'.*

**---Response**：Thank you. All above-mentioned files were submitted.

*Please note while forming your response, if your article is accepted, you may have the opportunity to make the peer review history publicly available. The record will include editor decision letters (with reviews) and your responses to reviewer comments. If eligible, we will contact you to opt in or out.*

**---Response**：Thank you. We are willing to make the peer review history publicly available.

*Journal Requirements:*

*When submitting your revision, we need you to address these additional requirements.*

*1. Please ensure that* *your manuscript meets PLOS ONE's style requirements, including those for file naming. The PLOS ONE style templates can be found at*

*http://www.journals.plos.org/plosone/s/file?id=wjVg/PLOSOne_formatting_sample_main_body.pdf and* *http://www.journals.plos.org/plosone/s/file?id=ba62/PLOSOne_formatting_sample_title_authors_affiliations.pdf*

**---Response**：Thank you. We have proofread our manuscript carefully to meet PLOS ONE's style requirements.

*2. We understand that no formal ethical approval was obtained for this research even though it included the participation of 20 tasters.*

*Please clarify if your* *institutional review board (IRB)* *waived the need to obtain formal approval and consent from participants. If participant consent was obtained, please* *amend your methods section to state this and specify whether the consent was informed and if verbal or written.*
**---Response**：We appreciate this comment. Our institutional review board don’t need to obtain formal approval and consent from participants and all tasters have known that the evaluate results may be published. We added the sentence “All tasters evaluated the fruits objectively and agreed to publish their evaluation results.” at methods section.

*Reviewers' comments:
Reviewer's Responses to Questions*
***Comments to the Author*** *1. Is the manuscript technically sound, and do the data support the conclusions?
The manuscript must describe a technically sound piece of scientific research with data that supports the conclusions. Experiments must have been conducted rigorously, with appropriate controls, replication, and sample sizes. The conclusions must be drawn appropriately based on the data presented. 
Reviewer #1: Yes*

*2. Has the statistical analysis been performed appropriately and rigorously? 
Reviewer #1: Yes*

*3. Have the authors made all data underlying the findings in their manuscript fully available?
The* [*PLOS Data policy*](http://www.plosone.org/static/policies.action#sharing) *requires authors to make all data underlying the findings described in their manuscript fully available without restriction, with rare exception (please refer to the Data Availability Statement in the manuscript PDF file). The data should be provided as part of the manuscript or its supporting information, or deposited to a public repository. For example, in addition to summary statistics, the data points behind means, medians and variance measures should be available. If there are restrictions on publicly sharing data—e.g. participant privacy or use of data from a third party—those must be specified.
Reviewer #1: Yes*

*4. Is the manuscript presented in an intelligible fashion and written in standard English?
PLOS ONE does not* *copyedit accepted manuscripts, so the language in submitted articles must be clear, correct, and unambiguous. Any typographical or grammatical errors should be corrected at revision, so please note any specific errors here.
Reviewer #1: No*

**---Response**：Thank you. We appreciate this comment and checked the whole manuscript seriously. We have also asked a colleague who is a native English speaker to carefully proofread the manuscript. We believe that all grammar problems have been corrected.

*5. Review Comments to the Author
Please use the space provided to explain your answers to the questions above. You may also include additional comments for the author, including concerns about dual publication, research ethics, or publication ethics. (Please upload your review as an attachment if it exceeds 20,000 characters)
Reviewer #1: Comments
1. The manuscript* *presents some interesting information that may be published after necessary revision.*

**---Response**：We appreciate this comment and have carefully revised our manuscript.

*2. Generally document read well but requires language editing before publication, the word grafting and grafted are used in a misappropriate way, please check. Similarly there are several other language mistake and formatting errors, please check carefully*

**---Response**：We appreciate this comment. In revised manuscript, the word grafting and grafted were used in an appropriate way. We have carefully checked each sentence in the manuscript and corrected all language mistake and formatting errors. For example:

L18. “The cause of bitterness produced after grafting” was replaced with “The cause of bitterness of fruits on grafted plants”.

L198 “In order to further analyze the fruits metabolites, the orthogonal partial least-squares discrimination analysis (OPLS-DA) models were established.” was replaced with “To further analyze the metabolites of fruits, OPLS-DA models were established.”

L463. “Information of different grafted rootstocks” was replaced with “Information of different rootstocks”.

*3. Please check the following
L 1.* *The word “grafting” should be replaced with “grafted”*

*L 24-25. “For example, bitter fruits sometimes occurred on grafted plants.” Replace with “**For example, sometimes bitter fruits are produced on grafted plants.”
L 164-166. Please check the word “ion”, this do not seems appropriate word for description of metabolites figure.*

**---Response**：We appreciate this comment and revised as suggested.

L 1. The word “grafting” was replaced with “grafted”

L 24-25. “For example, bitter fruits sometimes occurred on grafted plants.” was replace with “For example, sometimes bitter fruits are produced on grafted plants.”

L 164-166. “Total ion current maps” was replaced with “Base peak intensity (BPI) chromatograms” after referring to other researches.

*4. Please add a conclusion sentence at the end of abstract.*

**---Response**：We appreciate this comment and added the conclusion sentence “In summary, these results showed that the bitter fruits of grafted Balengcui were caused by *Cucurbita maxima* Duch. rootstocks. Phospholipids, cucurbitacins, and flavonoids were the key contributors for the occurrence of bitter fruits in Balengcui melon after grafting onto *Cucurbita maxima* Duch. rootstocks.” at the end of abstract.

*5. L 55. Following recent references may be cited
1. Mu Xiong, Xuejun Zhang, Sergey Shabala, Lana Shabala, Yanjun Chen, Chengli Xiang, Muhammad Azher Nawaz, Zhilong Bie, Haibo Wu, Hongping Yi, Mingzhu Wu, Yuan Huang. 2018.* *Evaluation of salt tolerance and contributing ionic mechanism in nine Hami melon landraces in Xinjiang, China. Scientia Horticulturae. 237:277-286.
https://www.sciencedirect.com/science/article/pii/S0304423818302723
2. Muhammad Azher Nawaz, Muhamamd Imtiaz, Qiusheng Kong, Cheng Fei, Waqar Ahmed, Yuan Huang and Zhilong Bie. (2016). Grafting: a technique to modify ion accumulation in horticultural crops. Frontiers in Plant Science. 7: 1457. (IF: 4.298). http://journal.frontiersin.org/article/10.3389/fpls.2016.01457/full
3. Zhilong Bie, Muhammad Azher Nawaz, Yuan Huang，Jung-Myung Lee and Giuseppe Colla. 2017. Introduction to Vegetable Grafting. In. Giuseppe Colla, Francisco Perez Alfocea, Dietmar Schwarz (Eds.). Vegetable Grafting. Principles and Practices. CABI Publishing, UK. pp. 1-21.*

**---Response**：We appreciate this comment and cited above-mentioned references as suggested.

*6. L 57.* *“Grafted on” should be replaced with “grafted onto”, please check and replace this throughout the manuscript.*
**---Response**：Thank you. We replaced “grafted on” with “grafted onto” in revised manuscript and checked the whole manuscript carefully as suggested.

*7. Discussion section requires improvement.*
**---Response**：We appreciate this comment. Discussion section was modified and improved in revised manuscript.

*8. Conclusion section just seems a summary of results, please improve and add a conclusive statement and future aspects of research in this field.*
**---Response：**We appreciate this comment and improved the conclusion section. Especially, We add a conclusive statement “These results indicated that during summer cultivation, oxidation and decomposition of phospholipids affected the fruits of plants grafted onto pumpkin rootstocks and the methylovalerate pathway was activated to synthesize cucurbitacins.”and future aspects of research in this field “This study enables a better understanding of the mechanisms of how bitter fruits are produced on plants grafted on specific rootstocks. Grafting onto pumpkin rootstocks for summer production should be avoided and other special rootstocks should be selected and bred.” in Conclusion section.

*9. Please supply a high quality Figures particularly Figure 1.*

**---Response**：We appreciate this comment and high quality Figures were supplied in revised manuscript.

*6. PLOS authors have the option to publish the peer review history of their article (*[*what does this mean?*](https://journals.plos.org/plosone/s/editorial-and-peer-review-process#loc-peer-review-history)*). If published, this will include your full peer review and any attached files.
If you choose “no”, your identity will remain anonymous but your review may still be made public.*
***Do you want your identity to be public for this peer review?*** *For information about this choice, including consent withdrawal, please see our* [*Privacy Policy*](https://www.plos.org/privacy-policy)*.
Reviewer #1: Yes: Muhammad Azher Nawaz*

*While revising your submission, please upload your figure files to the Preflight Analysis and Conversion Engine (PACE) digital diagnostic tool,* [*https://pacev2.apexcovantage.com/*](https://pacev2.apexcovantage.com/)*. PACE helps ensure that figures meet PLOS requirements. To use PACE, you must first register as a user. Registration is free. Then, login and navigate to the UPLOAD tab, where you will find detailed instructions on how to use the tool. If you encounter any issues or have any questions when using PACE, please email us at* [*figures@plos.org*](mailto:figures@plos.org)*. Please note that Supporting Information files do not need this step.*

**---Response**：Thank you. All figure files were uploaded to PACE and no Image Problems were discovered. In revised manuscript, all figures were adjusted according to PACE Adjustments.
